# Branch Numbers and Crop Load Combination Effects on Production and Fruit Quality of Flat Peach Cultivars (*Prunus persica* (L.) Batsch) Trained as Catalonian Vase

**DOI:** 10.3390/plants11030308

**Published:** 2022-01-24

**Authors:** Luca Mazzoni, Irene Medori, Francesca Balducci, Micol Marcellini, Paolo Acciarri, Bruno Mezzetti, Franco Capocasa

**Affiliations:** 1 Department of Agricultural, Food and Environmental Sciences, Università Politecnica delle Marche, Via Brecce Bianche 10, 60131 Ancona, Italy; l.mazzoni@staff.univpm.it (L.M.); irenemedori@hotmail.it (I.M.); francesca.balducci@staff.univpm.it (F.B.); micol.marcellini@staff.univpm.it (M.M.); b.mezzetti@staff.univpm.it (B.M.); 2 Acciarri Società Agricola s.r.l., Via Aso 55, 63851 Ortezzano, Italy; paolo.acciarri@acciarri.eu

**Keywords:** crop load, sugars, acids, antioxidant capacity, polyphenols, yield, fruit size

## Abstract

Thinning and pruning are expensive cultural practices in peach cultivation, but essential to obtain adequate production. This study evaluated the effects of combining two pruning (four and six scaffold branches) and three thinning (low, medium, and high crop load) levels on yield and fruit quality of four different flat peach cultivars, trained as Catalonian vase in 2017–2018 in Italy. Productive (average fruit weight, plant total production, and fruit circumference), qualitative (fruit firmness and overcolor, Soluble Solids Content, and Titratable Acidity), and nutritional (Total Antioxidant Capacity, and Total Phenol Content) parameters were evaluated. For productive parameters, a high crop load level led to a decrease in fruit weight and circumference, while a high crop load resulted in higher plant yield. Regarding the qualitative parameters, fruit SSC significantly increased with the diminution of the crop load level in both years of study, while TA was not influenced by crop load and number of branches. Both the total antioxidant capacity and the polyphenol content decreased with an increase in branches number. The findings derived from this study will help growers to select the most suitable combination among genotypes and plant management, to obtain the desired productive or qualitative goals.

## 1. Introduction

The different training systems adopted worldwide for peach (*Prunus persica* (L.) Batsch) cultivation have different productive potentials depending on the cultivation conditions and the cultivation systems adopted. To better understand the different factors controlling tree performances and fruit quality, a great number of experiments have been performed since 1950 [1]. Environmental, genetic, and agronomic parameters are the main factors responsible for the variation in plant yield and fruit characteristics in fruit species, including peach. The fruit maturity at harvest strongly influences the peach market life and quality, so the definition of the correct harvest time is fundamental. The cultivar/rootstock combination is also a key determinant of the fruit quality and the plant yield in peach [2,3] and many breeding programs are aiming to create new genotypes with improved productivity and quality attributes [4,5]. Mineral fertilization and irrigation are cultivation factors that have been studied for many years, and their effects on peach fruit quality and plant production have been highlighted [4,6]. Furthermore, the relation among canopy management, fruit quality, and plant yield has been studied in different combinations [7,8,9]. Light interception and its adequate distribution within the canopy are the primary factors determining high yield of high quality and homogenous fruit. Therefore, the orchards that managed to promote the highest homogeneity in canopy light interception are promoting high plant yield of high-quality fruit [3,10]. Plant yield and fruit quality are also strictly related to the appropriate crop load, depending on the type of training system adopted. For this reason, the definition and management of the most efficient canopy training system, depending on the cultivar and rootstock/cultivar interaction, is critical for reaching the highest fruit quality and uniformity at harvest [11,12]. Many studies have analyzed the effect of peach canopy management on plant yield and fruit commercial quality, such as size, soluble sugars, and color, but not many studies are available on specific sensorial fruit quality, especially regarding nutritional compounds [13].

Thinning is a very expensive and labor-intensive practice for peach growers, as it is usually performed by hand. Many studies are searching for alternative thinning methods to reduce this labor, and a suitable solution could be chemical thinning. Although plant growth regulators are established thinning practices in other fruit crops (apples and pears), there are few similar products available for peach that promote abscission of flowers or fruits [14]. Anyhow, thinning is essential for a better control of the crop load and for reaching the largest fruit size and improved fruit quality, the two most important traits taken into consideration by the peach market [15,16].

Different studies showed how crop load can influence fruit size, harvesting time, and fruit quality [17] and, in particular, a high crop load may slow down the ripening process and, consequently, postpone the harvesting time [18,19]. Furthermore, the thinning timing could influence the following fruit growth response. Thinning at bloom stage greatly minimizes the effects of competition among fruit, thereby maximizing growth potential of the fruit [20,21]. However, such early crop-load management strategies can potentially result in excessive reduction of fruit number per tree as some retained flowers may not set fruit, natural abscission could take place, or adverse weather could limit fruit set, leading to dramatic yield losses [22,23,24,25]. Fruit thinning before pit hardening (during S1) is a commonly used strategy in peach crop-load management to minimize these negative aspects of bloom thinning while allowing for increases in fruit size [26,27]. Another less labor-intensive alternative is thinning after natural abscission, as only excess fruit need removal [28], but resource limitations during early stages severely impact fruit growth [20,29]. The crop load must be considered in combination with the rootstock/cultivar and canopy training system. This equilibrium should comprise also the climatic conditions that can occur particularly during the blossom and the fruit set period. The most appropriate crop load is also important to distribute fruits near the photosynthetic organs, hence fruits may absorb photosynthesis products more easily [30]. Fruit position within the canopy influences fruit size, red overcolor, and ripening time [4,7,31,32], as well as sensorial and nutritional parameters such as antioxidant capacity and total phenolic content; these latter aspects are closely linked with the red skin overcolor [3]. Solid soluble content decreases downwards, beginning from the highest layers of the canopy towards the lower layers, regardless of the training system or the rootstock. This is probably due to the lower light interception in the lower canopy layers [3,33], even if a study hypothesizes that this effect is due to hormonal signals related to the fruit position [34]. Furthermore, fruit position along the shoot presents high relevance; basal fruit has higher solid soluble concentration than the distal fruit along the same shoot [35,36].

In recent years, a new training system, the Catalonian vase, obtained great success in Spain and then in different areas of peach and other stone fruit cultivation, whereby it led to a significant reduction in production costs, mostly for the reduced labor costs, achieved for the easier tree management [37]. This type of training system is fully managed from the ground, because trees present a low height; therefore, they do not need temporary or permanent supports. The branch number is not determined: it may vary from more open forms, consisting of four to five branches, to a denser shape, consisting of six to eight branches or even more [38,39,40].

In peach cultivation, in recent years, there has been a wide spread of new flat fruit cultivars. For these cultivars it is essential to manage the most appropriate crop load to reach the largest size, without the risk of fruit cracking, and to improve the quality of the fruit.

Many studies have analyzed pruning and thinning effects on plant production and fruit quality, but the interaction between these two practices has not been elucidated. The main aim of the present study is to describe the best pruning and thinning combination to apply for increasing peach fruit sensorial and nutritional quality in four flat peach cultivars trained as Catalonian vase, without negatively affecting the plant yield.

## 2. Results and Discussion

The five tested cultivars showed different blooming and harvesting times.

Regarding blooming (Table 1), the earliest cultivars were Galaxy and Platibelle, also resulting as more exposed to late frost risks. Blooming began more than 10 days later for Plane^®^ Delicious and Plane^®^ Star cultivars. Average blooming beginning and lasting periods corresponded to what is reported in the literature for flat peaches, which are expected to start blooming at around 6–7 March and last for 18–20 days [41]. In particular, Platibelle, which is considered a mid-blooming cultivar, showed an ending blooming time similar to what is indicated in the literature (16–20 March) [42], but with some days of difference between 2017 and 2018.

These differences in blooming time corresponded to a difference in harvesting time. Galaxy was harvested earlier compared to the other studied cultivars (Table 2), as the ripening occurred within the first 10 days of July. Plane^®^ Star was the last cultivar to be harvested, as ripening took place between the third and the fourth weeks of August. Harvest was carried out two or three times in the season, depending on how fast fruit ripening occurred. In both 2017 and 2018, fruits of the cultivars Galaxy and Plane^®^ Delicious were harvested on three dates, while Platibelle and Plane^®^ Star on two harvest dates.

### 2.1. Yield Parameters

Variance analysis, related to the average fruit weight, total tree yield, and fruit circumference, indicates that productive parameters were statistically influenced by the year in which the trial was carried out, by the crop load level and also by the cultivar (Table 3).

The results indicate a clear difference among the two cultivation years, mostly due to the different climatic conditions during blooming—fruit set period. In particular, in 2017, these conditions caused a different fruit set among the genotypes, but it was possible to maintain the same amount of crop load between the two pruning levels for each variety.

The different number of branches did not influence any of the yield parameters, despite several studies indicating that the training system and pruning have an effect on tree yield due to different light interception of the canopy [43,44]. The crop load significantly affects the productive parameters (average fruit weight, tree total production, fruit circumference), as confirmed by other studies [20]. Regarding the combination of the different variables of the study, the most affected parameter was the average fruit weight, while tree total production and fruit circumference were influenced only by particular combinations. There is no significant influence of the interaction between number of branches and years of study on the productive parameters, while the most effective combination of factors is Year∗Cultivar∗Crop load, which influenced all productive parameters with high significance.

#### 2.1.1. Tree Total Yield

The statistical analysis shows that crop load strongly influenced tree total yield (Table 4). For all cultivars tested, there was an increasing productive trend from a low crop load toward a high crop load. The cultivar Galaxy maintained this increasing trend with increasing crop load, but the yields at medium and high crop loads were not significantly different. Excluding Galaxy, all of the other cultivars showed significant differences among crop load levels. The same conclusion was reached by Njoroge and Reighard [22] for the cv Contender, while Drogoudi et al. [45] did not find any significant difference among three different levels of thinning in the peach cultivar Andross. In our study, both Plane^®^ cultivars were the most productive at higher crop load. At lower crop loads, there were no significant productive differences among cultivars.

#### 2.1.2. Average Fruit Weight

Crop load induced differences in the average fruit weight (Table 4). The effect is of different amplitude according to the different flat peach cultivars, but the general trend is that a low crop load allowed to obtain fruits with increased weights (an average of about 20 g more than the other two crop loads). Interestingly, no fruit cracking problems were noted, although it was demonstrated that, in some cultivars, fruits tend to crack when plants are thinned too much [4]. Our results on flat peach cultivars are in line with many studies in the literature: e.g., Berman and DeJong [46] demonstrated that an increased crop load caused a reduction in the average fruit weight in the peach cultivar Elegant Lady, while Inglese et al. [47] reached the same results for Early May Crest and Flaminia cvs. Additionally, in Alcobendas et al. [48], the low crop load led to a higher average fruit weight than the “commercial” load in cv. Flordastar, but the difference was not significant. In our study, the highest average fruit weight was obtained in 2018 due to the larger structure of the tree, almost four years old, that had completed canopy growth, thus better supporting the crop load (less competition between vegetative and productive activity) with respect to year 2017.

#### 2.1.3. Fruit Circumference

The different crop loads had different effects on fruit circumference according to the cultivar considered: in any case, these differences were not significant (Table 4). The trend showed that fruit circumference is larger for low crop loads and decreased moving to higher crop loads. These differences are less evident in 2017 than in 2018. Many studies have already demonstrated that fruit size increases with lower crop loads, as shown in Alcobendas et al. [48] and in Njoroge and Reighard [22] for fruit diameter. Besides the amount of fruit per plant, also distance between fruit has been reported to influence the fruit size [49,50], but Marini [51] stated that fruit size is dependent on the number of fruit per tree, irrespective of the distance between fruits. The genotype effect is more evident than crop load by far, with Galaxy presenting the biggest fruits, and Plane^®^ Star the smallest ones.

### 2.2. Qualitative Parameters

Variance analysis, related to the fruit firmness, overcolor, Soluble Solids Content, and Titratable Acidity, indicates that all of the qualitative parameters were statistically influenced only by the cultivar, while year and crop load influenced, to different extents, the qualitative parameters (Table 5).

Only the interaction Year∗Cultivar was able to exert a significant effect on all parameters related to fruit quality. The TA was not affected by any other interaction. Regarding the overcolor, it was significantly affected only by Year∗Cultivar and Year∗Crop load interactions. Firmness and SSC changed significantly, besides Year∗Cultivar interaction, with Cultivar∗Crop load and Year∗Cultivar∗Crop load interactions. Shown in Table 5, the Cultivar effect is strongly affecting the fruit quality, and its interaction with year and crop load could influence some of the analyzed parameters in a significant manner.

#### 2.2.1. Fruit Firmness

This parameter is strongly influenced by year, cultivar, and the interaction between these two factors (Table 5), but it is not influenced by crop load. This is not in line with several studies in which crop load significantly influenced fruit ripening [13,17]. According to Table 6, only in Plane^®^ Delicious was a significant effect of crop load on fruit firmness detected, with the low crop load producing firmer fruits than medium crop load. In general, the trend seems opposite for the other cultivars, with fruit produced under low crop loads being less firm than under higher load. However, these differences are minimal. Instead, cultivar effect is evident, with Plane^®^ Star presenting the firmest fruits at all crop loads.

#### 2.2.2. Fruit Overcolor

This parameter was influenced by the cultivar, the crop load, by the interaction between these two factors, and by Year∗Cultivar interaction (Table 5), while it was not influenced by the year of cultivation and the number of branches. This result was unexpected, because light distribution within the canopy was reported to strongly influence fruit skin overcolor, as shown by other researchers [50,52]. In our study, the typical open shape of the training system adopted, the Catalonian vase, allowed a good light penetration within the canopy also with a higher number of branches, avoiding excessive shading. This could have prevented excessive skin overcolor differences between fruit harvests on four-branched and six-branched trees. Table 6 clearly shows that crop load did not significantly influence the fruit overcolor, while the genotype effect is evident: in particular, the cultivar Platibelle had the significantly highest skin overcolor.

#### 2.2.3. Soluble Solids Content (SSC)

This parameter was influenced by the crop load, the year of the study, the cultivar, and by some interactions among these factors (Table 5). The high crop load level caused a significant decrease in the average fruit SSC compared to the low crop load level in all the tested cultivars ((Table 7). These results agree with other previous studies in which it was reported that thinning reduces the competition for photosynthesis products among fruits [20]. Therefore, a lower crop load level leads to a higher accumulation of photosynthetic products in the fruits, also causing an increase in SSC [53]. In our study, the genotype effect is also evident, with Plane^®^ Star producing fruits with the significantly highest SSC at each crop load level. The other three cultivars were significantly similar to each other at all crop load levels.

#### 2.2.4. Titratable Acidity (TA)

The fruit TA was influenced only by Cultivar and the interaction Year∗Cultivar (Table 5). These findings are in accordance with the results shown in Table 7 where a non-significant effect of crop load on fruit acidity is evident (only Galaxy revealed a significant difference between low crop load and the other two crop load levels). The genotype effect is clear, with Galaxy and Platibelle having the most acidic fruits, followed by Plane^®^ Star and finally Plane^®^ Delicious.

### 2.3. Nutritional Parameters

Nutritional parameters data are available only for the year 2018. Total phenolic content (TPH) and total antioxidant capacity (TAC) were both influenced by cultivar, crop load, and by number of branches, and by all their interactions, except n° of Branches∗Crop load (Table 8). The TAC and the TPH (Table 9) were affected by the crop load, but the trend and the extent of the variation was related to the different cultivar considered. Furthermore, the branch number also affected the nutritional parameters, decreasing in fruits harvested on plants pruned at six branches with respect to plants pruned at four branches. Therefore, number of branches and the crop load influenced fruit nutritional composition. This result can be explained by the strong influence of canopy architecture on the light interception, which is connected to the skin red overcolor and to the synthesis of specific nutritional compounds such as anthocyanins [3]. According to Table 9, fruits of Galaxy and Plane^®^ Star obtained in high crop load-trees presented lower levels of both TAC and TPH with respect to low crop load-trees. These results are in agreement with Drogoudi et al. [45], where TPH and TAC were found to be greater in fruits from heavily thinned in comparison to lightly or moderately thinned trees. Contrarily, in Buendia et al. [54], the crop load did not influence the content of different phenolic compounds detected in fruits of the cv Flordastar. These results were confirmed in our study by Plane^®^ Delicious and Platibelle, which presented similar values of TPH in fruits harvested from plants subjected to different crop loads (Table 9).

### 2.4. Principal Component Analysis (PCA)

**Figure 1:**
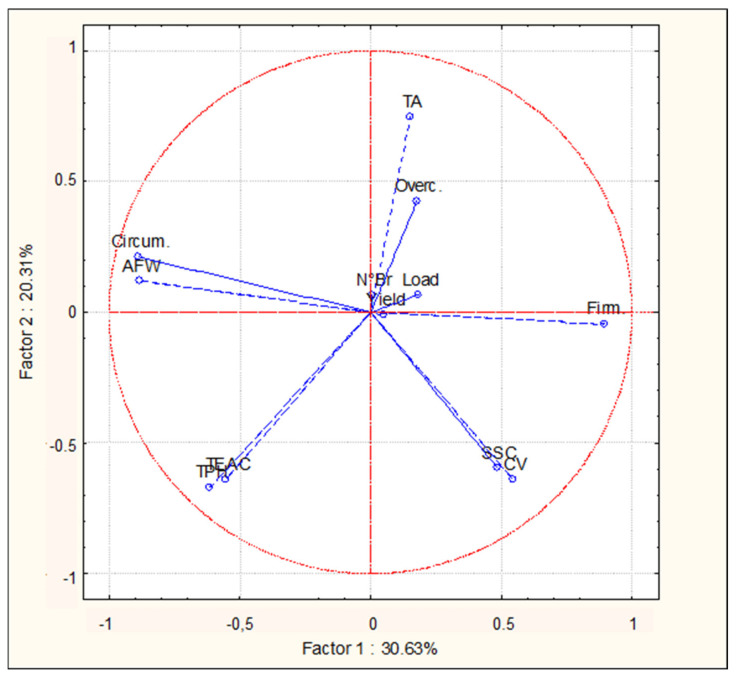
Bi-plot graph of the productive, qualitative, and phytochemical parameters analyzed in this study, and the variables considered (vector distribution). Factors 1 and 2 explain 50.94% of the data variation.

The PCA bi-plot of productive, qualitative, and nutritional parameters showed interesting results, highlighting a common trend for some of the parameters that were analyzed (Figure 1). The spectrophotometric phytochemical parameters (TPH and TEAC) were in the same quadrant (lower left). Similarly, overcolor and Titratable Acidity (TA) were together on the upper right quadrant, opposite to the quadrant of the nutritional parameters. This is quite surprising, given that fruit overcolor is due to an increase in the amount of colored pigment, which belong to secondary metabolites (such as anthocyanins) that also possess antioxidant capacity. This finding suggests that antioxidant capacity in fruits of the analyzed peaches is more related to other not-colored compounds, probably mainly present in the pulp of the fruit. Average fruit weight (AFW) and fruit circumference were in the same higher left quadrant, and this was expected, meaning that bigger fruits showed an higher weight. Opposite to this quadrant, in the lower right part of the graph, we find the Soluble Solids Content (SSC) and the firmness, even if their vectors are not as close each other. This result suggests that fruits with lower dimensions (circumference and weight), tend to be firmer and sweeter. Regarding the distribution of the variables on the bi-plot plan, there are also some interesting results. The Cultivars (CV) vector is very close to the SSC vector: this result suggests that, among the evaluated parameters, the SSC is the most affected by the CV effect, being influenced mostly by this parameter rather than n° of branches (N°Br) and Crop load (Load). These two variables are found in the middle part of the graph, indicating that they are not influencing only one parameter. However, the “Yield” parameter is placed very close to crop load vector, indicating that this parameter is mostly affected by the crop load (as also indicated in Table 3).

## 3. Materials and Methods

### 3.1. Plant Material and Experimental Sites

**Figure 2:**
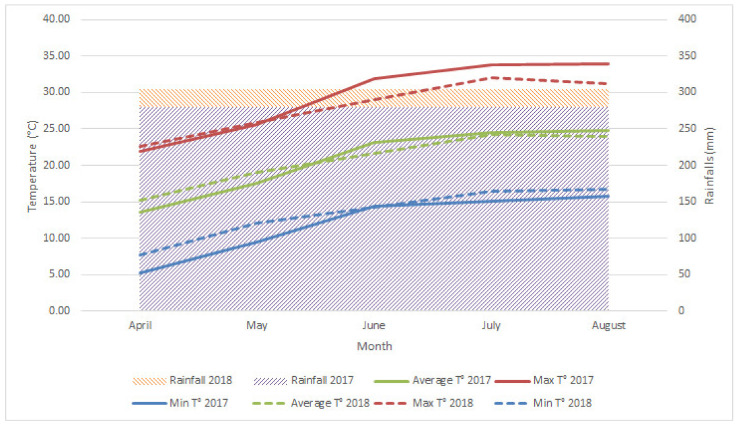
Average rainfall in the 2017 and 2018 seasons, and average monthly minimum, medium, and maximum temperatures in the 2017 and 2018 seasons in the orchard.

**Figure 3:**
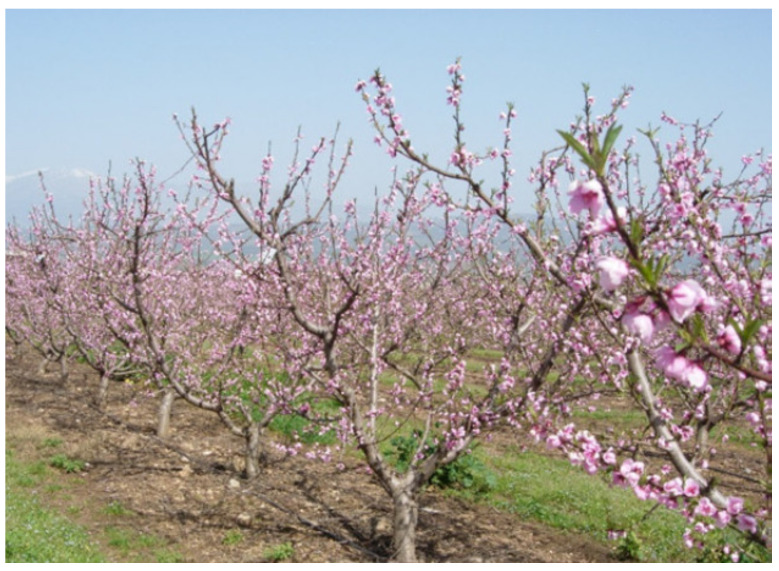
Example of the Catalonian vase training system (adapted from Neri et al., 2015 [55]).

The study was carried out in a commercial peach orchard planted in 2014, in Marche Region, Italy (43.012344N, 13.362602E). The experiment was conducted over two years (2017 and 2018), in an orchard positioned in a valley floor area. In the April–August period of 2017 and 2018 seasons, temperatures and rainfall were measured with a weather station (iMetos-Pessl Instruments, Weiz, Austria) (Figure 2). Temperature ranges from 5.3 to 33.9 °C, and average rainfall was 279.6 and 304.4 mm in the 2017 and 2018 seasons, respectively. The research was carried out on four flat peach cultivars, with white flesh (Galaxy, Platibelle, Plane^®^ Delicious and Plane^®^ Star), with different bloom and harvest periods, grafted on Garnem^®^ rootstock, and spaced 5 × 3 m. The ‘Catalonian vase’ training system was applied for all trees (Figure 3). All the agronomic practices applied to the orchard followed the integrated production specification of the Marche Region (https://www.regione.marche.it/Portals/0/Agricoltura/ProduzioneIntegrata/DDS_AFP_2012_0016.pdf, accessed on 16 February 2016).

The experimental trial was set up to compare two pruning models: pruning retaining six scaffold branches and pruning retaining four scaffold branches. These two pruning models were combined with three crop load levels (low, medium, high) (Table 10). The experimental design was set up in three randomized blocks containing six trees per block (2 pruning models × 3 crop load levels) for each cultivar; each tree represented a parcel (18 trees in total) (Figure S1). In the two-years study (2017 and 2018), trees were at the 4th and 5th cultivation cycles: this means that they were in full production only during the second season, while in 2017 the canopy growth was not completed yet. In both years, a thinning operation was carried out manually between late April and early May, just before the hardening of the fruit kernel (about 40 days after full blooming). The number of fruit left on trees was decided in order to have the same number of fruits per area of tree, with increasing values moving from low to high crop level. In the first year of study, fruit set was very different among cultivars, and the number of fruits per crop load changed according to the cultivars, ranging from a minimum of 85 fruits/tree to a maximum 320 fruits/tree. In the following year (2018), fruit set was very similar among cultivars, and it was possible to homogenize the number of fruits for low, medium, and high crop load among all the cultivars, from a minimum of 100 fruits/tree to a maximum of 400 fruits/tree (Table 10). Harvesting was carried out two or three times per season, depending on the ripening time of the cultivars and the optimal commercial maturation (firmness of 4.0 ± 0.5 kg). Thirty fruits positioned in the middle external part of the canopy were sampled from each tree and used for determining the qualitative and nutritional parameters.

### 3.2. Productive Parameters

#### 3.2.1. Average Fruit Weight

Thirty fruits were collected from each tree during each harvest period and weighed on a digital scale (Orma-Milano). Data were expressed in grams (g).

#### 3.2.2. Tree Total Production

The total yield per tree at each harvest period was weighed on a digital dynamometer (Kern CH 50K50) and the weight was expressed in kilograms (kg).

#### 3.2.3. Fruit Circumference

Thirty fruits were sampled from each tree at each harvest and measured with a commercial caliber. The fruit circumference was expressed in centimeters (cm).

### 3.3. Qualitative Parameters

At each harvest, 30 fruits were harvested for each tree. The fruits were divided in three replicates of 10 fruits each. For each replicate, the following parameters were analyzed.

#### 3.3.1. Fruit Firmness

Harvesting time was established based on the flesh firmness, which was measured using a manual penetrometer (Turoni, Forlì, Italy) with an 8-mm diameter tip. The fruits sampled at each harvest were perforated, after the removal of the peel, in 2 diametrically opposed points. Data were expressed in kilograms (kg).

#### 3.3.2. Fruit Overcolor

The skin overcolor of the fruits sampled was measured with visual evaluation and expressed in percentage of overcolor on total skin surface (%).

#### 3.3.3. Soluble Solids Content

Fruit Soluble Solids Content was measured with a digital temperature compensation refractometer N-1E (Atago, Tokyo, Japan). At each harvest, the juice was extracted with a centrifuge (BOSCH, Stuttgart, Germany). From this juice, one or two drops were dropped on the refractometer prism for reading. The measure was expressed as °Brix.

(a) Titratable Acidity

Fruit Titratable Acidity was determined from 10 mL of the same juice extracted for the Soluble Solids Content analysis, diluted with distilled water, and titrated with 0.1 N NaOH solution, until pH 8.2, and expressed as % of Malic Acid Equivalents (% MAE).

### 3.4. Nutritional Parameters

Total Antioxidant Capacity (TAC) and Total Phenolic Content (TPH) were measured on fruit samples after a methanolic extraction performed on 10 selected fruits per tree per harvest, cut in two specular slices, then minced into small pieces, weighed (10 g) and added to 100 mL of methanolic solution (20:80 water:methanol and 1% of acetic acid). Samples were homogenized using an Ultraturrax T25 homogenizer (Janke and Kunkel, IKA Labortechnik, Staufen, Germany). The homogenized suspensions were stored in a fridge at 4 °C in the dark. After 48 h, the suspensions were centrifuged at 2000 rpm for 20 min (Rotofix32 centrifuge, Hettich Zentrifugen, Tuttlingen, Germany) and the recovered supernatants were collected and stored in individual amber vials, of 2 mL each. These vials were stored at −20 °C until the day of analysis [56,57].

#### 3.4.1. Total Antioxidant Capacity (TAC)

This parameter was evaluated through the Trolox Equivalent Antioxidant Capacity (TEAC) method [58,59,60]. A glass test-tube was filled with 1.9 mL of 2,2′-azino-bis(3-ethylbenzothiazoline-6-sulfonic acid) (ABTS). Afterwards, 0.1 mL of the diluted extract (1:10) was added and the solution was stored in the dark for 6 min. Then, the absorbance of the sample was measured at 734 nm through a spectrophotometer. The TAC is expressed as mM Trolox eq/kg fruit. The calibration was calculated by linear regression from the dose–response curve of the Trolox standards.

#### 3.4.2. Total Phenol Content (TPH)

This parameter was evaluated through the Folin–Ciocalteu method [61], with gallic acid as the standard for the calibration curve. Briefly, glass test-tubes were filled with 7.0 mL of water and 1 mL of ethanolic extract previously diluted (1:3). This step was followed by the addition of 500 µL of Folin–Ciocalteu reagent, swirling the samples. After 3 min, 1.5 mL of sodium carbonate (0.53 mol/L) were added, and the test-tubes were mixed again and then stored in the dark for 60 min. After that, the absorbance of the samples was measured at 760 nm. Data were calculated and expressed as mg gallic acid per kg of fruit (mg GA/kg fruit).

### 3.5. Statistical Analysis

All the productive, qualitative, and nutritional parameters were analyzed using the multivariate test analysis of variance (ANOVA), to determinate differences among different years, branch number, crop load, cultivars, and their interactions. Significant differences within samples were calculated according to Fisher tests (Least Significant Difference, LSD). Principal component analysis (PCA) was also used to evaluate the levels of association among the productive, qualitative, and nutritional parameters, and among the variables “cultivars”, “crop load”, and “n° of branches”. In the graph, the parameters and the variables that are closest to each other in the same geometric plane of the bi-plot are considered to be interrelated, and consequently the parameters and the genotypes that are distant from each other are not related or are negatively related. The greater the distance of a vector from the origin of the axis, the higher the correlation of the variable with the PC represented in that axis. All analyses were performed using the software STATISTICA 7.0 (StatSoft. Tulsa, OK, USA). Differences were considered significant for *p* ≤ 0.05.

## 4. Conclusions

Thinning and pruning in the peach orchard are expensive but necessary practices to obtain adequate and high-quality production also for flat peach cultivars. The results of this study highlight that, for these peach cultivars, the crop load affects all productive, qualitative, and nutritional parameters, while the number of branches influenced only the nutritional parameters. For both productive and qualitative parameters, there were significant influences observed between the two years of study. This might have been because in 2017 the tree growth had not yet completely formed, whereas in 2018 the tree architecture was complete and able to better support fruit growth. It is fundamental to underline that the genotype strongly influenced all the yield and quality parameters. Different cultivars behaved differently when subjected to the same pruning and thinning levels, demonstrating that the choice of the right cultivar/tree management combination is crucial to obtain the expected productive or qualitative performances.

We are conscious that these results could be better confirmed by taking into consideration more aspects, such as the inhomogeneity of plants in the two years of study, the possibility to correlate results with more detailed climatic parameters, the possibility to include in the study other cultivars, and/or other pruning models different from the Catalonian vase.

This study is a preliminary investigation on the effect of the simultaneous application of thinning and pruning on the production and quality of flat peach cultivars. These practices resulted very helpful to ameliorate the fruit quality and can be applied to develop the most appropriate management systems for any new flat or rounded peach cultivars.

## Figures and Tables

**Table 1:** Blooming progress in the four cultivars. Dates registered during the years 2017 and 2018 (dd/mm/yy).

Year	Cultivar	Blooming Beginning (10% Open Flowers)	Full Blooming (50% Open Flowers)	Blooming Ending (90% Open Flowers, Begin of Petal Fall)
2017	Galaxy Platibelle Plane^®^ Delicious Plane^®^ Star	03/03/17 03/03/17 16/03/17 14/03/17	10/03/17 07/03/17 20/03/17 18/03/17	19/03/17 14/03/17 25/03/17 24/03/17
2018	Galaxy Platibelle Plane^®^ Delicious Plane^®^ Star	05/03/18 07/03/18 19/03/18 18/03/18	12/03/18 15/03/18 25/03/18 24/03/18	20/03/18 19/03/18 03/04/18 02/04/18

**Table 2:** The harvesting times of the four cultivars studied. Dates registered during the years 2017 and 2018 (dd/mm/yy).

Year	Cultivar	1° Harvest	2° Harvest	3° Harvest
2017	Galaxy	3/07/17	6/07/17	10/07/17
	Platibelle	11/07/17	14/07/17	
	Plane^®^ Delicious	25/07/17	28/07/17	2/08/17
	Plane^®^ Star	18/08/17	21/08/17	
2018	Galaxy	5/07/18	9/07/18	12/07/18
	Platibelle	16/07/18	19/07/18	
	Plane^®^ Delicious	25/07/18	30/07/18	2/08/18
	Plane^®^ Star	17/08/18	22/08/18	

**Table 3:** Multivariate test analysis (ANOVA). Data refers to average fruit weight, tree total production, fruit circumference. ** = significant differences for *p* < 0.01; * = significant differences for *p* < 0.05; n.s. = non-significant differences.

Factor	Average Fruit Weight	Tree Total Production	Fruit Circumference
Year (a)	**	**	**
Cultivar (b)	**	**	**
n° Branches (c)	n.s.	n.s.	n.s.
Crop load (d)	**	**	**
Year∗Cultivar (a × b)	**	**	**
Year∗n° Branches (a × c)	n.s.	n.s.	n.s.
Year∗Crop load (a × d)	*	**	**
Cultivar∗n° Branches (b × c)	**	n.s	n.s
Cultivar∗Crop load (b × d)	n.s.	*	*
n° Branches∗Crop load (c × d)	**	n.s.	n.s.
Year∗Cultivar∗n° Branches (a × b × c)	*	n.s.	*
Year∗Cultivar∗Crop load (a × b × d)	**	**	**
Year∗n° Branches∗Crop load (a × c × d)	**	n.s.	n.s.
Cultivar∗n° Branches∗Crop load (b × c × d)	*	*	*
Year∗Cultivar∗n° Branches∗Crop load (a × b × c × d)	*	n.s.	*

**Table 4:** Influence of the n° of branches and the crop load on average fruit weight (g/fruit), yield (kg/tree), and fruit circumference (cm) of different cultivars in 2017 and 2018. Data are expressed as mean ± standard errors. Data of the same parameter with different lowercase letters are significantly different (*p* ≤ 0.05). Mean values of cultivars with different uppercase letters are significantly different (*p* ≤ 0.05). Mean values of years with the asterisk are significantly different (*p* ≤ 0.05). LSD Test. GX: Galaxy; PB: Platibelle; PD: Plane® Delicious; PS: Plane® Star.Cultivar.

	n° Branches	Crop Load	Average Fruit Weight	Yield	Fruit Circumference
GX 2017			124.45 ± 2.43 C	31.39 ± 1.02 A	24 ± 0.04 C
	4	low	139.58 ± 4.33 ghijk	27.08 ± 1.09 lmn	24.36 ± 0.1 def
	4	medium	125.36 ± 3.68 jklmnopqr	32.01 ± 1.25 ghijk	24.24 ± 0.1 efg
	4	high	119.48 ± 3.39 klmnopqrs	36.01 ± 1.08 efg	23.96 ± 0.11 hi
	6	low	127.01 ± 5.83 jklmnopqr	26.33 ± 2.09 lmno	23.84 ± 0.12 ij
	6	medium	116.68 ± 6.02 klmnopqrst	31.03 ± 1.21 hijkl	23.58 ± 0.11 jk
	6	high	118.56 ± 2.88 klmnopqrst	35.88 ± 0.68 efgh	24.04 ± 0.11 ghi
PB 2017			110.63 ± 3.04 CD	15.48 ± 1.31 C	22.59 ± 0.06 F
	4	low	121.59 ± 5.47 klmnopqrs	7.54 ± 1.4 x	22.83 ± 0.14 qrst
	4	medium	102.63 ± 2.78 rstuvw	15.27 ± 0.64 stu	22.37 ± 0.15 uv
	4	high	99.04 ± 0.77 stuvw	21.14 ± 0.82 pqr	22.27 ± 0.14 vw
	6	low	129.93 ± 0.42 ijklmnop	10.35 ± 0.67 vwx	23.33 ± 0.14 klmn
	6	medium	112.11 ± 1.67 nopqrstuv	16.64 ± 1.43 qrstu	22.7 ± 0.11 stu
	6	high	98.49 ± 3.12 stuvw	21.92 ± 0.63 op	22.03 ± 0.16 vwx
PD 2017			118.54 ± 7.29 C	22.32 ± 1.6 B	23 ± 0.04 E
	4	low	136.18 ± 2.54 hijklmn	16.28 ± 0.14 rstu	23.27 ± 0.1 lmnop
	4	medium	81.95 ± 40.98 w	20.59 ± 0.49 pqrst	23 ± 0.09 opqrs
	4	high	120.65 ± 5.13 klmnopqrs	32.94 ± 0.82 fghi	22.98 ± 0.09 opqrs
	6	low	134.23 ± 1.31 hijklmno	15.07 ± 0.5 uv	23.16 ± 0.08 mnopqr
	6	medium	125.97 ± 0.85 jklmnopqr	21.29 ± 0.48 pq	22.93 ± 0.08 qrst
	6	high	112.28 ± 1.24 mnopqrstuv	27.14 ± 0.69 klmn	22.67 ± 0.1 tu
PS 2017			102.63 ± 2.27 D	26.21 ± 0.85 AB	22.25 ± 0.06 G
	4	low	112.82 ± 0.61 lmnopqrstu	22.83 ± 1.45 nop	23.03 ± 0.12 nopqrs
	4	medium	103.61 ± 3.52 qrstuvw	28.48 ± 0.87 ijklm	21.97 ± 0.13 wx
	4	high	89.82 ± 1.26 uvw	27.29 ± 0.8 klmn	21.43 ± 0.12 y
	6	low	105.9 ± 4.5 pqrstuvw	21.33 ± 1.49 pq	22.63 ± 0.14 tu
	6	medium	109.46 ± 5.18 opqrstuv	28.83 ± 2.05 ijklm	22.63 ± 0.14 tu
	6	high	94.17 ± 1.72 tuvw	28.5 ± 0.55 ijklm	21.8 ± 0.14 x
Total 2017			114.06 ± 2.31	23.87 ± 0.92	23.07 ± 0.03
GX 2018			189.11 ± 5.6 A	20.71 ± 2.28 BC	24.97 ± 0.01 A
	4	low	216.67 ± 9.62 a	8.57 ± 0.97 x	25 ± 0 a
	4	medium	187.11 ± 10.65 bcd	21.92 ± 1.95 op	24.98 ± 0.02 ab
	4	high	169.64 ± 3.04 cdef	27.88 ± 1.02 jklm	24.93 ± 0.04 ab
	6	low	191.86 ± 0.42 abc	8.7 ± 0.68 wx	24.98 ± 0.02 ab
	6	medium	178.27 ± 8.29 cde	24.88 ± 3.01 mnop	25 ± 0 a
	6	high	191.09 ± 26.12 bc	32.28 ± 1.5 ghij	24.96 ± 0.03 ab
PB 2018			158.15 ± 6.22 B	29.26 ± 2.18 A	24.06 ± 0.07 BC
	4	low	165.78 ± 2.93 def	16.68 ± 1.44 qrstu	25 ± 0 ab
	4	medium	139.86 ± 1.12 ghijk	32.53 ± 0.71 fghij	24.23 ± 0.14 efgh
	4	high	121.52 ± 2.97 klmnopqrs	39.83 ± 4.03 cde	23.17 ± 0.19 mnopqr
	6	low	163.78 ± 2.18 defg	20.2 ± 1.01 pqrst	24.6 ± 0.1 bcd
	6	medium	154.72 ± 4.05 efghi	28.93 ± 2.8 ijklm	24.57 ± 0.11 cde
	6	high	203.24 ± 5.19 ab	37.4 ± 1.02 def	23.27 ± 0.17 klmnop
PD 2018			153.93 ± 4.91 B	31.72 ± 3.08 A	24.14 ± 0.06 B
	4	low	181.56 ± 2.82 bcde	15.15 ± 0.4 stuv	24.9 ± 0.06 abc
	4	medium	156.12 ± 5.53 efgh	34.6 ± 1.11 fgh	24.22 ± 0.11 fgh
	4	high	137.29 ± 3.85 hijkl	44.77 ± 2.29 ab	23.58 ± 0.16 jk
	6	low	172.06 ± 5.96 cdef	13.5 ± 2.53 uvw	24.83 ± 0.07 abc
	6	medium	157.92 ± 2.04 efgh	35.4 ± 0.62 efgh	24.38 ± 0.1 def
	6	high	127.82 ± 9.83 jklmnopq	41.4 ± 4.14 bcd	23.4 ± 0.14 klm
PS 2018			122.57 ± 5.72 C	28.78 ± 3.35 A	23.23 ± 0.08 D
	4	low	149.4 ± 6.37 fghij	15.75 ± 0.92 stu	24.37 ± 0.13 def
	4	medium	122.86 ± 4.87 klmnopqrs	29.17 ± 0.91 ijklm	23.53 ± 0.14 jkl
	4	high	116.35 ± 2.97 klmnopqrst	42.83 ± 2.26 bc	22.37 ± 0.18 uv
	6	low	137.2 ± 4.73 hijklm	14.28 ± 0.75 uv	23.8 ± 0.17 ij
	6	medium	87.82 ± 18.86 vw	21.07 ± 4.5 pqr	23.3 ± 0.16 klmnop
	6	high	121.8 ± 11.33 klmnopqs	49.57 ± 3.21 a	22 ± 0.18 wx
Total 2018			155.97 ± 3.96 *	27.56 ± 1.44 *	24.2 ± 0.03 *

**Table 5:** Multivariate test analysis (ANOVA). Data referred to flesh firmness, skin overcolor, Soluble Solid Content (SSC), Titratable Acidity (TA). ** = significant differences for *p* < 0.01; * = significant differences for *p* < 0.05; n.s. = non-significant differences.

Factor	Firmness	Overcolor	SSC	TA
Year (a)	**	n.s.	**	n.s.
Cultivar (b)	**	**	**	**
n° Branches (c)	n.s.	n.s.	n.s.	n.s.
Crop load (d)	n.s.	*	**	n.s.
Year∗Cultivar (a × b)	**	**	**	**
Year∗n° Branches (a × c)	*	n.s.	*	n.s.
Year∗Crop load (a × d)	n.s.	*	**	n.s.
Cultivar∗n° Branches (b × c)	n.s.	n.s.	n.s.	n.s.
Cultivar∗Crop load (b × d)	*	n.s.	*	n.s.
n° Branches∗Crop load (c × d)	n.s.	n.s.	n.s.	n.s.
Year∗Cultivar∗n° Branches (a × b × c)	n.s	n.s.	n.s.	n.s.
Year∗Cultivar∗Crop load (a × b × d)	*	n.s.	*	n.s.
Year∗n° Branches∗Crop load (a × c × d)	n.s	n.s.	n.s.	n.s.
Cultivar∗n° Branches∗Crop load (b × c × d)	n.s.	n.s.	n.s.	n.s.
Year∗Cultivar∗n° Branches∗Crop load (a × b × c × d)	n.s.	n.s.	n.s.	n.s.

**Table 6:** Influence of the n° of branches and the crop load on fruit firmness (kg) and overcolor (%) of different cultivars in 2017 and 2018. Data are expressed as mean ± standard errors. Data of the same parameter with different lowercase letters are significantly different (*p* ≤ 0.05). Mean values of cultivars with different uppercase letters are significantly different (*p* ≤ 0.05). Mean values of years with the asterisk are significantly different (*p* ≤ 0.05). LSD Test. GX: Galaxy; PB: Platibelle; PD: Plane^®^ Delicious; PS: Plane^®^ Star.

Cultivar	n° Branches	Crop Load	Firmness	Overcolor
GX 2017			4.19 ± 0.02 BC	80.5 ± 0.41 D
	4	low	4.32 ± 0.05 cdef	80.89 ± 0.98 jklmno
	4	medium	4.17 ± 0.04 ghij	78.44 ± 0.91 opqr
	4	high	4.25 ± 0.04 defgh	82.94 ± 1.15 ghijkl
	6	low	4.13 ± 0.04 ghijk	80.61 ± 1.03 klmno
	6	medium	4.14 ± 0.04 ghijk	79.39 ± 0.93 mnopq
	6	high	4.11 ± 0.05 hijk	80.72 ± 1.03 klmno
PB 2017			4.38 ± 0.02 A	85.94 ± 0.46 B
	4	low	4.57 ± 0.05 a	87.17 ± 1 bcde
	4	medium	4.49 ± 0.05 ab	85.67 ± 1.03 cdefg
	4	high	4.24 ± 0.06 defgh	83.67 ± 0.99 efghijk
	6	low	4.35 ± 0.05 bcde	87.33 ± 1.21 abcde
	6	medium	4.36 ± 0.05 bcde	84.83 ± 1.23 cdefghi
	6	high	4.25 ± 0.05 defgh	87 ± 1.24 bcde
PD 2017			4.14 ± 0.02 C	74.74 ± 0.44 F
	4	low	4.1 ± 0.05 ijk	71 ± 1.19 x
	4	medium	4.24 ± 0.04 defgh	73.5 ± 1.46 uvwx
	4	high	4.14 ± 0.05 ghijk	77.11 ± 0.73 pqrs
	6	low	4.15 ± 0.04 ghijk	73.5 ± 1.13 uvwx
	6	medium	4.06 ± 0.04 jkl	76.56 ± 0.77 pqrst
	6	high	4.16 ± 0.04 ghij	76.78 ± 0.78 pqrst
PS 2017			4.32 ± 0.03 A	83.85 ± 0.48 C
	4	low	4.24 ± 0.07 defgh	84.92 ± 1.15 cdefghi
	4	medium	4.44 ± 0.06 abc	84.17 ± 1.35 defghij
	4	high	4.43 ± 0.07 abc	83.83 ± 1.24 efghijk
	6	low	4.2 ± 0.06 efghij	82.25 ± 1.03 ghijklm
	6	medium	4.37 ± 0.07 bcd	84.83 ± 1.21 cdefghi
	6	high	4.27 ± 0.06 defg	83.08 ± 1.07 fghijkl
Total 2017			4.24 ± 0.01 *	80.53 ± 0.25 *
GX 2018			3.59 ± 0.02 E	78.06 ± 0.5 E
	4	low	3.46 ± 0.06 r	80.5 ± 1.13 klmno
	4	medium	3.73 ± 0.04 pq	77.17 ± 1.12 pqrs
	4	high	3.77 ± 0.04 opq	71.89 ± 1.11 wx
	6	low	3.55 ± 0.04 r	78.22 ± 1.35 opqr
	6	medium	3.48 ± 0.04 r	83.22 ± 1.21 fghijk
	6	high	3.53 ± 0.03 r	77.33 ± 1.16 pqrs
PB 2018			3.86 ± 0.02 D	87.98 ± 0.55 A
	4	low	3.64 ± 0.07 qr	90.67 ± 1.67 ab
	4	medium	4.01 ± 0.05 klm	91 ± 0.83 a
	4	high	3.75 ± 0.05 opq	86.42 ± 1.51 bcdef
	6	low	3.84 ± 0.06 nop	87.67 ± 1.33 abcd
	6	medium	3.89 ± 0.04 mno	88 ± 1.35 abc
	6	high	3.92 ± 0.04 lmn	85.5 ± 1.23 cdefgh
PD 2018			3.9 ± 0.02 D	77.03 ± 0.51 E
	4	low	4.1 ± 0.07 hijk	76.42 ± 1.36 pqrstu
	4	medium	3.76 ± 0.05 opq	81.67 ± 0.88 ijklmn
	4	high	3.89 ± 0.05 mno	74.67 ± 1.08 stuvw
	6	low	4.12 ± 0.07 ghijk	76 ± 1.55 pqrstu
	6	medium	3.94 ± 0.05 lmn	78.83 ± 1.21 nopqr
	6	high	3.76 ± 0.06 opq	74.06 ± 1.31 tuvw
PS 2018			4.22 ± 0.03 B	77.11 ± 0.7 E
	4	low	4.12 ± 0.07 ghijk	81.83 ± 1.3 hijklmn
	4	medium	4.2 ± 0.06 efghi	79.58 ± 1.64 lmnopq
	4	high	4.19 ± 0.07 fghij	77.08 ± 1.82 pqrst
	6	low	4.23 ± 0.07 defghi	76.27 ± 1.79 pqrstu
	6	medium	4.26 ± 0.06 defg	75.67 ± 1.79 rstuv
	6	high	4.35 ± 0.06 bcde	72.25 ± 1.62 vwx
Total 2018			3.86 ± 0.01	79.49 0.3

**Table 7:** Influence of the n° of branches and the crop load on Soluble Solids Content (° Brix) and Titratable Acidity (% malic acid equivalents) of different cultivars in 2017 and 2018. Data are expressed as mean ± standard errors. Data of the same parameter with different lowercase letters are significantly different (*p* ≤ 0.05). Mean values of cultivars with different uppercase letters are significantly different (*p* ≤ 0.05). Mean values of years with the asterisk are significantly different (*p* ≤ 0.05). LSD Test. GX: Galaxy; PB: Platibelle; PD: Plane^®^ Delicious; PS: Plane^®^ Star.

Cultivar	n° Branches	Crop Load	Soluble Solids Content	Titratable Acidity
GX 2017			12.64 ± 0.09 D	4 ± 0.08 A
	4	low	12.94 ± 0.18 ijklm	3.96 ± 0.14 abcd
	4	medium	12.51 ± 0.11lmnopq	4 ± 0.23 abcd
	4	high	12.23 ± 0.08 pqrs	4.17 ± 0.21 a
	6	low	13.58 ± 0.1 efgh	3.88 ± 0.21 abcde
	6	medium	12.36 ± 0.22 nopqrs	4.08 ± 0.23 abc
	6	high	12.2 ± 0.09 qrs	3.91 ± 0.16 abcde
PB 2017			13.28 ± 0.08 C	4.13 ± 0.03 A
	4	low	13.52 ± 0.12 efgh	4.23 ± 0.1 a
	4	medium	13.32 ± 0.2 ghij	4.2 ± 0.03 a
	4	high	12.93 ± 0.1 ijklmn	4.13 ± 0.07 abc
	6	low	13.78 ± 0.16 efg	4.03 ± 0.11 abcd
	6	medium	13.18 ± 0.07 hij	4.17 ± 0.1 ab
	6	high	12.95 ± 0.22 ijklm	4.03 ± 0.07 abcd
PD 2017			13.4 ± 0.07 C	3.26 ± 0.04 D
	4	low	13.73 ± 0.17 efg	3.17 ± 0.13 jkl
	4	medium	13.21 ± 0.17 hij	3.41 ± 0.07 fghijk
	4	high	13.01 ± 0.14 ijk	3.31 ± 0.04 hijkl
	6	low	13.63 ± 0.12 efgh	3.11 ± 0.13 kl
	6	medium	13.47 ± 0.19 gh	3.3 ± 0.16 ijkl
	6	high	13.37 ± 0.13 ghi	3.28 ± 0.08 ijkl
PS 2017			15.38 ± 0.08 A	3.25 ± 0.06 D
	4	low	15.58 ± 0.13 ab	3.22 ± 0.1 jkl
	4	medium	15.07 ± 0.23 bc	2.98 ± 0.11 l
	4	high	15.38 ± 0.1 ab	3.24 ± 0.21 ijkl
	6	low	15.28 ± 0.27 ab	3.54 ± 0.16 efghij
	6	medium	15.63 ± 0.17 a	3.32 ± 0.08 ghijkl
	6	high	15.33 ± 0.26 ab	3.22 ± 0.11 jkl
Total 2017			13.54 ± 0.08 *	3.65 ± 0.04
GX 2018			12.85 ± 0.09 D	3.7 ± 0.06 C
	4	low	13.56 ± 0.11 efgh	3.31 ± 0.13 hijkl
	4	medium	12.68 ± 0.13 klmnop	3.64 ± 0.11 defghi
	4	high	12.12 ± 0.14 qrs	3.98 ± 0.1 abcd
	6	low	13.54 ± 0.08 efgh	3.67 ± 0.17 defgh
	6	medium	12.86 ± 0.18 jklmno	3.91 ± 0.08 abcde
	6	high	12.37 ± 0.09 nopqrs	3.73 ± 0.18 bcdef
PB 2018			11.8 ± 0.07 E	3.8 ± 0.05 BC
	4	low	12.27 ± 0.15 nopqrs	3.87 ± 0.21 abcdef
	4	medium	12.12 ± 0.08 qrs	3.8 ± 0.15 abcdef
	4	high	11.47 ± 0.15 t	3.73 ± 0.14 bcdefg
	6	low	11.95 ± 0.08 rst	3.87 ± 0.1 abcde
	6	medium	11.85 ± 0.22 st	3.78 ± 0.16 abcdef
	6	high	11.45 ± 0.07 t	3.78 ± 0.12 abcdef
PD 2018			11.97 ± 0.08 E	3.11 ± 0.04 D
	4	low	12.37 ± 0.23 nopqrs	3.04 ± 0.09 kl
	4	medium	12.11 ± 0.24 qrs	3.09 ± 0.1 kl
	4	high	11.57 ± 0.09 t	3.12 ± 0.08 kl
	6	low	12.42 ± 0.15 lmnopqr	3.03 ± 0.13 kl
	6	medium	12.18 ± 0.12 qrs	3.04 ± 0.06 kl
	6	high	11.48 ± 0.1 t	3.3 ± 0.17 ijkl
PS 2018			13.69 ± 0.18 B	3.96 ± 0.06 AB
	4	low	14.58 ± 0.32 cd	4 ± 0.14 abcd
	4	medium	14.07 ± 0.48 de	4.08 ± 0.17 abc
	4	high	13.25 ± 0.17 ghij	3.72 ± 0.14 cdefg
	6	low	14 ± 0.21 ef	3.82 ± 0.1 abcdef
	6	medium	13.78 ± 0.54 efg	4.03 ± 0.11 abcd
	6	high	12.48 ± 0.25 lmnopqr	4.09 ± 0.22 abc
Total 2018			12.59 ± 0.08	3.61 ± 0.04

**Table 8:** Multivariate test analysis (ANOVA). Data referred to Total Phenolic Content (TPH) and Total Antioxidant Capacity (TAC). ** = significant differences for *p* < 0.01; * = significant differences for *p* < 0.05; n.s. = non-significant differences.

Factor	TPH	TAC
**Cultivar (a)**	**	**
**n° Branches (b)**	**	**
**Crop load (c)**	**	*
**Cultivar∗n° Branches (a × b)**	**	**
**Cultivar∗Crop load (a × c)**	**	**
**n° Branches∗Crop load (b × c)**	n.s.	n.s.
**Cultivar∗n° Branches∗Crop load (a × b × c)**	**	**

**Table 9:** Influence of the n° of branches and the crop load on Total Antioxidant Capacity (mM Trolox eq/kg fruit) and Total Phenolic Content (mg GA/kg fruit) of different cultivars in 2018. Data are expressed as mean ± standard errors. Data of the same parameter with different lowercase letters are significantly different (*p* ≤ 0.05). Mean values of cultivars with different uppercase letters are significantly different (*p* ≤ 0.05). LSD Test. GX: Galaxy; PB: Platibelle; PD: Plane^®^ Delicious; PS: Plane^®^ Star.

Cultivar	n° Branches	Crop Load	Total Antioxidant Capacity	Total Phenolic Content
GX			8.97 ± 0.25 A	1041 ± 25.71 A
	4	low	10.87 ± 0.14 a	1276 ± 21.71 a
	4	medium	8.66 ± 0.29 ab	1036 ± 20.03 cd
	4	high	7.66 ± 0.33 bc	934 ± 26.08 de
	6	low	9.68 ± 0.31 ab	1051 ± 33.84 bcd
	6	medium	10.35 ± 0.45 a	1154 ± 58.42 b
	6	high	6.63 ± 0.41 cd	798 ± 26.14 fg
PB			3.49 ± 0.25 D	528 ± 24.13 D
	4	low	5.03 ± 0.08 defghi	600 ± 1.91 jk
	4	medium	4.85 ± 0.75 efghi	670 ± 42.93 hij
	4	high	4.44 ± 0.39 fghij	609 ± 7.8 j
	6	low	1.82 ± 0.07 l	411 ± 82.05 l
	6	medium	2.21 ± 0.45 kl	422 ± 49.46 l
	6	high	3.39 ± 0.22 hijk	490 ± 25.51 kl
PD			6.99 ± 0.29 B	847 ± 20.23 B
	4	low	6.09 ± 0.19 de	809 ± 11.01 fg
	4	medium	8.83 ± 0.37 ab	1054 ± 32.3 bc
	4	high	7.64 ± 0.23 bc	843 ± 36.75 ef
	6	low	5.59 ± 1.2 def	750 ± 63.72 fghi
	6	medium	5.43 ± 0.13 defg	818 ± 28.43 efg
	6	high	8.34 ± 0.22 ab	807 ± 33.19 fg
PS			4.67 ± 0.28 C	725 ± 22.1 C
	4	low	4.18 ± 0.25 ghij	710 ± 9.25 ghij
	4	medium	4.87 ± 0.48 efghi	649 ± 22.65 ij
	4	high	3.83 ± 0.7 hij	706 ± 50.23 ghij
	6	low	6.63 ± 1.05 cd	867 ± 101.8 ef
	6	medium	5.29 ± 0.56 defg	776 ± 31.64 fgh
	6	high	3.24 ± 0.18 jk	643 ± 9.4 ij
Total 2018			6.09 ± 0.2	791 ± 17.71

**Table 10:** Combinations between two pruning models (branch number) and three thinning levels (crop load) in four flat peach cultivars. Data related to the two-year period of 2017 and 2018.

Year	Branch Number	Crop Load Level	Fruit Number/Tree			
			Galaxy	Platibelle	Plane^®^ Delicious	Plane^®^ Star
**2017**	6	low	200	85	120	220
		medium	260	150	170	270
		high	300	210	240	320
	4	low	200	85	120	220
		medium	260	150	170	270
		high	300	210	240	320
**2018**	6	low	100	100	100	100
		medium	250	250	250	250
		high	400	400	400	400
	4	low	100	100	100	100
		medium	250	250	250	250
		high	400	400	400	400

## Supplementary Materials

The following supporting information can be downloaded at: www.mdpi.com/article/10.3390/plants11030308/s1, Figure S1: Experimental design of the study.
