# Supplementary material for: Branch Numbers and Crop Load Combination Effects on Production and Fruit Quality of Flat Peach Cultivars (Prunus persica (L.) Batsch) Trained as Catalonian Vase
Source: Plants (Basel). 2022 Jan 24;11(3):308. doi: 10.3390/plants11030308 (PMC8839559; doi:10.3390/plants11030308)
Supplement: Supplementary file 1 [file plants-11-00308-s001.zip › plants-1537329-supplementary.pdf]

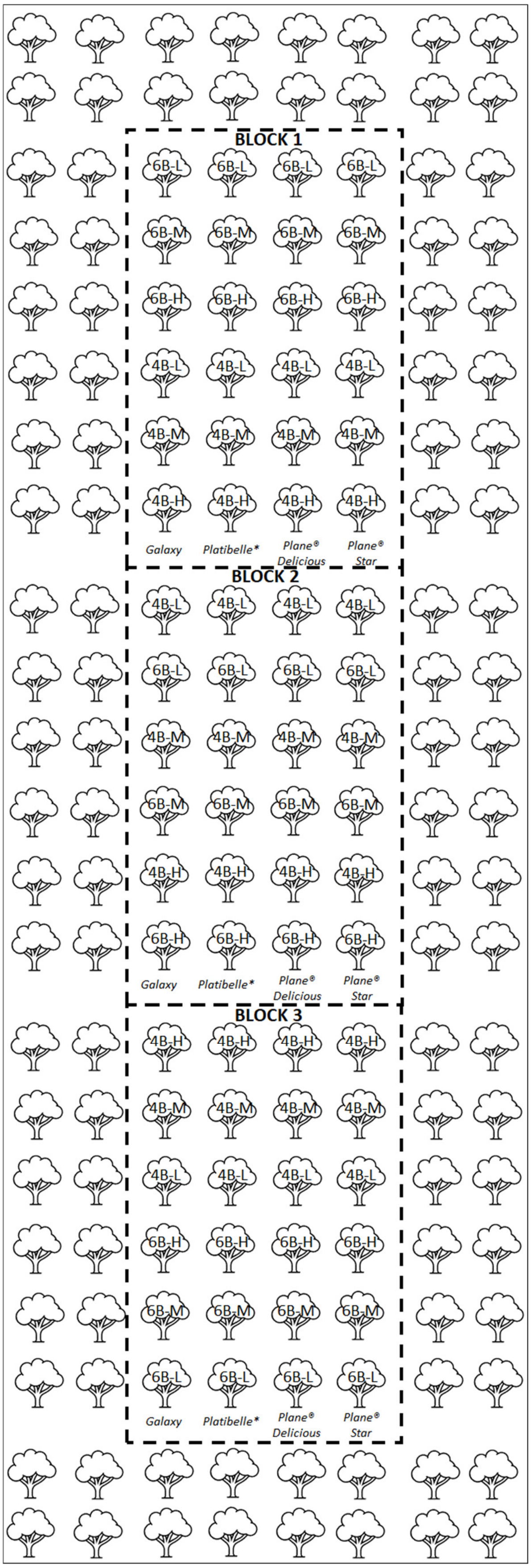

Figure S1. Experimental design of the study (4B: 4 branches; 6B: 6 branches; L: low crop load; M: medium crop load; H: high crop load). This scheme was used for both 2017 and 2018 years. the dotted edges delimit the study area. The trees outside this area represent the “board” effect.
